# Effectiveness of a kindergarten-based intervention to increase vegetable intake and reduce food neophobia amongst 1-year-old children: a cluster randomised controlled trial

**DOI:** 10.29219/fnr.v65.7679

**Published:** 2021-10-08

**Authors:** Eli Anne Myrvoll Blomkvist, Andrew K. Wills, Sissel Heidi Helland, Elisabet Rudjord Hillesund, Nina Cecilie Øverby

**Affiliations:** 1 Department of Public Health, Sport and Nutrition, Faculty of Health and Sport Sciences, University of Agder, Kristiansand, Norway; 2 Faculty of Health Sciences, University of Bristol, Bristol, UK

**Keywords:** children, kindergarten, food neophobia, vegetables, sensory education, Sapere, web-based, online resources

## Abstract

**Background:**

Children’s first years of life are crucial to their future health. Studies show that a varied diet with a high intake of vegetables is positive in several domains of health. The present low vegetable intake amongst children is, therefore, a concern. Food neophobia is a common barrier to vegetable intake in children. As most Norwegian children attend kindergarten from an early age, kindergartens could contribute to the prevention of food neophobia and the promotion of vegetable intake.

**Objective:**

The aim of this study was to assess the effect of a cluster randomised trial amongst 1-year-old children in kindergarten to reduce food neophobia and promote healthy eating.

**Methods:**

Kindergartens were randomly allocated to either a control group or one of two intervention groups. Both intervention groups (diet and diet + Sapere-method) were served a warm lunch meal including three alternating intervention vegetables, whilst the intervention group 2 (diet + Sapere) in addition received tools for weekly sensory lessons. The intervention was digitally administered via information and recipes on a study website. The control group did not receive any information. Parents completed digitally distributed questionnaires addressing food neophobia and food habits at baseline and post-intervention.

**Results:**

The parents of 144 1-year-old children in 46 kindergartens completed the questionnaires, which were included in the main analysis. The results suggested a higher intake of the intervention vegetables in group 2 (diet + Sapere) compared to the control group. The effect on total vegetable intake was inconclusive. No effect was observed on the level of food neophobia in either of the intervention group.

**Conclusion:**

This digitally delivered dietary and sensory intervention promoted the intake of intervention-targeted vegetables with inconclusive effect on total vegetable intake due to large loss to follow-up. No effect on the level of food neophobia was detected.

## Popular scientific summary

- A low vegetable intake amongst children is of concern, and the kindergarten is a promising setting to increase this intake.
- We have evaluated a web-based intervention in kindergarten, aiming to improve child diet by increasing vegetable intake and reducing food neophobia using a randomised controlled design.
- The main intervention elements were kindergarten staff serving vegetable-containing lunch dishes to the children and implementing weekly sensory lessons.
- Results indicate that such an intervention may improve intake of some vegetables; however, we found no effect on food neophobia.

What we eat has a significant impact on health and disease throughout the life course (1, 2). A low intake of fruits and vegetables increases the risk for non-communicable diseases and mortality (3–7). Despite what we know about the health benefits of diets rich in fruits and vegetables, the intake of these food groups is too low in many countries (8). The average intake of fruit and vegetables in 1-year-old children in Norway is lower than the recommendations, especially regarding vegetables (9, 10).

One barrier for vegetable intake in children can be food neophobia. Neophobia literally means ‘fear of the new’, and food neophobia is defined as an unwillingness to eat unfamiliar foods (11). Food neophobia is considered a normal developmental stage that typically starts when the child is around 2 years old. It is most explicit in children between 2 and 6 years of age and gradually decreases with age into a relatively stable level in adulthood (12). Food neophobia is negatively associated with food variety and may lead to an inadequate nutrient intake (13–16). Perry et al. (13) and Bell et al. (17) highlight the need to expose children to a wide variety of nutritious foods before the age of 2, which is the age when food neophobia tends to peak.

Repeated exposure, also known as mere exposure to foods, can increase a child’s liking and intake of a food. A recent review (18) found that repeated exposure is a simple and successful technique for increasing preschool children’s vegetable consumption. Studies find that as little as three to five exposures may be sufficient to increase food intake in young children (19–21).

Role modelling can also be efficient in influencing children’s food choices (22). Social cognitive theory suggests that modelling by teachers and peers is one of the most effective methods to encourage food acceptance in preschool children (23). Holley et al. (24) suggest that a combination of modelling, non-food rewards and repeated exposure is effective at increasing children’s consumption and liking of a previously disliked vegetable.

Sensory education could be a third way of influencing food acceptance. The aim of the sensory education is to awaken children’s curiosity and interest in foods, increase the willingness to taste new foods, and thereby potentially increase the intake of vegetables or other target foods in children (25, 26). One such sensory training method is the Sapere method, *sapere* meaning *to know*, *to feel*, *to taste*, based on Puisais’ work *Le Goût de L’enfant* (27). The Sapere method is used in both schools and kindergartens in other countries, amongst them Finland and Sweden (28–30). To our knowledge, the Sapere method has not been subject to research in kindergartens in Norway except from a trial done by our research group, in which Helland et al. (16, 31, 32) tested the Sapere method in children aged 2–3 years.

In Norway, more than 90% of all children between one and 5 years of age attend kindergarten, an educational service for children aged 0–5 years (33). In 2019, 84.4% of children in the age group of 1–2 years attended kindergarten. Most children eat three meals a day in kindergarten, which make up about 3,000–4,000 meals during his or her years in kindergarten (34). Meals are either brought from home (lunch boxes), provided by the kindergarten or a combination of the two. Few kindergartens have their own kitchen staff or cook, and the food served can vary widely (35). As the main meal, most kindergartens provide a cold meal with bread. Serving vegetables seems to be especially challenging; only one out of three kindergartens serves vegetables daily, whilst more than one in four kindergartens serves vegetables less often than once a week (35).

Food preferences and dietary patterns in early childhood can be tracked throughout childhood (36, 37). Young children tend to eat only what they like, but food preferences are modifiable through experimental learning or individual experience (38–40). Infants and toddlers are dependent upon parents and caregivers to feed them and are learning how to eat through familiarisation, observation and associative learning (41). The kindergarten setting is, thus, an arena with great opportunities to influence the food intake of young children.

Web-based intervention programmes designed to promote healthy eating can be both appealing, cost-effective and capable of reaching large groups of children and caregivers (42). There are various definitions of web-based interventions. For example, Koneska et al. defined web-based or a web intervention as ‘downloadable or accessible via the internet through a web browser’, which can take the form of (but not limited to) a website, an email or a web message board (43). Providing online resources and interactive tools represents a promising way of providing support to kindergartens and other types of childcare services (44).

The aim of the present study was to assess the effect of a web-based intervention with the purpose to promote a healthy and varied diet amongst 1-year-old children using a cluster randomised controlled design. In this paper, we report intervention effects on food neophobia and vegetable intake post-intervention.

## Methods

### Study design and participants

This study was designed as a cluster randomised controlled trial. The trial was registered in the ISRCTN Registry in May 2017, Trial registration number: ISRCTN98064772. In line with Norwegian guidelines for such research, the protocol for the present study was evaluated and approved by the Norwegian Centre for Research Data, 13 September 2016, reference 49951. An informed consent was obtained from parents of all participating children and from all kindergarten managers and participating kindergarten staff. The study protocol has been published elsewhere (45).

The recruitment of kindergartens started in May 2017, from all public and private kindergartens in four counties (Telemark, Oppland, Sør-Trøndelag and Møre og Romsdal) of Norway that met the following inclusion criterion (*n* = 1,043): having children of the appropriate age (i.e. born in 2016). The four counties covered two different geographical areas of Norway and included kindergartens located in both rural and urban settings. Kindergartens registered as ‘open kindergartens’ in which children and their parents attend together (*n* = 18), kindergartens registered with less than four children (*n* = 7) and kindergartens with children from 3 to 5 years only (*n* = 12) were not invited. The invitations were sent to the kindergarten managers by email and included detailed information about the study and a link to the study registration web page. The kindergarten managers got one reminder email after a couple of weeks. Because few kindergartens (*n* = 32) registered for the study initially, a random selection of kindergarten managers (*n* = 321) was additionally contacted by telephone and asked if they had received the email and further asked if they could be interested in participating in the study. The phone call recruitment lasted until the number of kindergartens registered was assumed to yield the planned study sample size.

Before randomisation, the pedagogical leaders in participating kindergarten departments were asked to distribute an electronic invitation letter to the parents of children born in 2016. The invitation provided detailed information about the study and a link to the registration web page where parents could register their child for the study. Parents were informed that they consented to participation by registering their child. Inclusion criteria included being born in the year of 2016 and at least one of the parents was able to read and understand Norwegian. Parents could register their child for the study from late August 2017 until the end of October, 2 weeks before the intervention started in November 2017. All included children turned 1 year during the year of 2017 when the intervention was carried out; hence, they named ‘one-year-olds’ throughout the paper. The baseline questionnaires were sent to parents by email shortly after registration and had to be completed electronically before randomisation.

The kindergartens were randomised to either the control group or one of two intervention groups (group 1: diet and group 2: diet + Sapere). The intervention period, a total of 12 weeks, lasted from November 2017 until February 2018. The post-intervention questionnaires were sent electronically immediately after the intervention period. The intervention kindergartens were given access to a password-protected study website with recipes and information videos developed and designed solely for this study. One of the main parts of the intervention was kindergarten staff serving vegetable-containing dishes to the children (see later under *Intervention*). However, all study contents, such as the recipes, instructions regarding the lunch serving and sensory lessons, educational material (videos) and the questionnaires, were delivered digitally; hence, it is defined as a web-based study.

### Sample size

The sample size was calculated according to the outcome food neophobia score. A previous cross-sectional analysis of a trial of 505 toddlers in Southern Norway (16) found a mean neophobia score of 18.2 (standard deviation [SD] = 9.3) amongst 2-year-old children. If a parent ticks on the lowest or second lowest alternative on the Child Food Neophobia scale (CFNS) for all the questions, it results in a CFNS score between 6 and 12, representing low levels of food neophobia (46). We, therefore, assumed that a mean score reduction in the level of food neophobia from 18.2 to 12.0 would be of public health relevance. A power of 80% and type 1 error of 5% suggested 36 participants in each group. To adjust for cluster variation, such standard sample size derived from formulas for individually randomised trials should be multiplied by the design effect, calculated using intra cluster-correlation coefficient (ICC) and numbers of participants in each cluster. As there was no ICC available for our purpose at the time of our project start, we estimated a value, as proposed by Ukoumunne et al. (47). To adjust for cluster variation, we therefore assumed an ICC of 0.1 in line with the literature, leading to a design effect factor of 1.6 expecting seven participants in each cluster (48, 49). Based on these calculations, we would need 58 participants in each group. Due to a probable loss to follow-up of participants of 20%, we aimed to recruit 70 children in each of the three groups, a total of 210 children for this study.

### Randomisation

The first author assigned each kindergarten a number according to when the kindergarten manager registered the kindergarten at the study web page. The first kindergarten to register got number 1, the second to register got number 2, etc. The 46 kindergartens included were randomised into one of three groups after the parents had completed the baseline questionnaire, approximately 2 weeks before the start of the intervention. The random allocation sequence was generated in SPSS by the last author, who had neither contact with the kindergartens nor access to or information from the completed questionnaires. The first author contacted the kindergarten managers to inform them about which group they were randomised to.

### Intervention

Two password-protected study websites, one for each of the two intervention groups, were developed by Aplia (aplia.no) in collaboration with university web-design personnel and the research group. The intervention *Barns matmot 2.0* was inspired by an earlier non-digital intervention targeting 2-year-old children in kindergarten, called *Barns matmot – Preschoolers Food Courage,* developed by Sissel H. Helland and co-workers (31). The purpose of *Barns matmot 2.0* was to develop a similar intervention adapted to 1-year-old children, before the onset of food neophobia, and to make all steps of the recruitment: the data collection and the information digital. Based on the experiences from the previous study *Barns matmot*, we also aimed to make the intervention *Barns matmot 2.0* somewhat simplified and less time-consuming for the kindergartens to implement (32). Kindergarten in both intervention groups were offered a compensation to buy necessary kitchenware, such as a good knife, saucepans or a hand blender. Only two kindergartens took advantage of this offer, suggesting that the kindergartens had the necessary equipment and facilities for food preparation. The intervention content is further described later.

No revision of intervention content was performed during the trial. If the kindergarten personnel had questions during the intervention, they could e-mail or telephone a contact person using information on the web site.

### Intervention element for both intervention groups

Children in both intervention groups were served a warm lunch meal with alternating vegetables for 3 days a week during the 3-month intervention period. The kindergartens had access to the three menus with nine different recipes in a password-protected website especially designed for each intervention group. Each of the three menus had one vegetable in focus, that is, spinach, celeriac and fennel (Table 1), hereby referred to as intervention vegetables. According to the Norwegian information bureau for fruit and vegetables, the most commonly used vegetables in Norway are tomato, carrots, onion, cucumber and bell pepper (50). The three intervention vegetables for this study were chosen to represent vegetables less commonly used in Norway to increase the probability that the vegetables were new to the children. A minimum of two meals per week included the intervention vegetables, so that the children were exposed to each vegetable at least six times during the menu period of 3 weeks. There was a 1-week ‘wash-out break’, where the kindergartens could serve their usual lunch meals between the three different menus. The parents of the registered children were also given access to the website with the nine recipes; however, the parents had no commitments or tasks regarding introducing the menus at home. Based on the experiences from the previous study *Barns matmot*, some of the recipes were simplified and others were replaced with less time-consuming recipes (32). The first author tested and revised all the recipes in advance to make them easily understandable and uncomplicated.

**Table 1:** Lunch dishes prepared in the intervention kindergartens

Menu	Vegetarian	Fish	Vegetarian
Menu 1 spinach	Pasta with vegetables and feta cheese (including spinach)	Pan fried fish with carrot purée	Spinach and lentils soup
Menu 2 celeriac	Celeriac soup	Salmon with celeriac purée	Vegetable stew (including celeriac)
Menu 3 fennel	Minestrone soup (including fennel)	Fish cakes with oven baked vegetables (including fennel)	Potato and broccoli omelette

### Additional intervention elements for intervention group 2

In addition to the lunch serving, the kindergarten staff in intervention group 2 (diet + Sapere) were instructed to implement pedagogical tools including weekly sensory lessons (Sapere method) (27) for the participating children and were given advice on meal practice and feeding practices during mealtime. During the sensory lessons, children were introduced to the intervention vegetable of the month, presented in three different ways; in the first week, it was presented raw, in the second week, raw with a dip and in the third week, it was presented differently (e.g. baked or otherwise prepared). In this way, children participating in the sensory lessons had three additional exposures of each food compared to intervention group 1, that is, at least nine exposures of the selected intervention vegetables. Recommendations for meal and feeding practices were presented in short informational videos on the study website that was only available to the kindergarten staff and parents in intervention group 2. The videos included information about food neophobia, repeated exposure, role modelling, our five senses, basic tastes and the Sapere method. The kindergarten staff were encouraged to sit down with the children and eat the same food during lunchtime.

Kindergartens in the control group were asked to continue their usual meal practices and did not get access to any information or web-based material.

### Outcomes and measures

Primary and secondary outcomes of the trial, as well as all measures and instruments, are presented in the study protocol (45). Only primary outcomes of the intervention are included in the present paper. The primary outcomes presented in this paper include child intake of intervention vegetables and all vegetables combined, and level of child food neophobia post-intervention.

To evaluate the effect of the two interventions on the given outcomes, parents completed digitally distributed questionnaires at baseline and post-intervention. A detailed description on how the outcomes were operationalised is provided later.

#### Vegetable intake

Child food intake was measured by selected items from a food frequency questionnaire (FFQ) that has been validated and used in large national surveys amongst 1- and 2-year-old children in Norway (9, 51–53). The frequency of intake was assessed without the specification of the amounts consumed. Questions on how often the child eats a broad selection of vegetables (e.g. ‘carrots’ or vegetable categories, such as ‘onions and leek’) were included, in addition to questions about fruits, berries, potatoes, bread and cereals, drinks, warm meals and snacks. The response options for the intake of vegetables and how they were re-coded into times per week were never = 0, <1/month = 0.1, 1–3/month = 0.5, 1–2/week = 1.5, 3–4/week = 3.5, 5–6/week = 5.5, 1/day = 7, 2/day = 14 and >3/day = 21.

The Norwegian Directorate of Health recommends at least five portions of fruits and vegetables per day preferably half (2.5 portions) should be vegetables, that is, 17.5 portions of vegetables per week (54, 55). The cut-off for desirable vegetable intake in our analysis was, therefore, set to 17.5 times per week to assess whether the interventions were effective in increasing the proportion of children that met the national guidelines for vegetable intake. The cut-off for desirable intake of the three intervention vegetables was set to a total of one time per week since they were quite uncommonly eaten; at baseline, only 17% were consuming at least one intervention vegetable per week and less than 6% were consuming at least two.

#### Child food neophobia

Child food neophobia was measured using a six-item version of Pliner’s 10-item CFNS (46). The CFNS is a validated tool that uses parental reporting of child food neophobia. The 6-item version of CFNS is commonly used to measure food neophobia in young children and has been used with children as young as 2 years (13, 16, 56, 57). The six items were (1) *My child is constantly sampling new and different foods* (reverse scored), (2) *My child does not trust new foods*, (3) *If my child doesn’t know what a new food is s(he) won’t try it*, (4) *My child is afraid to eat new things s(he) has never had before*, (5) *My child is very particular about the things s(he) eats* and (6) *My child will eat almost anything* (reverse scored). Responses were ranged from ‘strongly disagree’ (1) to ‘strongly agree’ (7) on a 7-point scale. A CFNS score was computed with higher scores indicating higher levels of food neophobia (range 6–42). The CFNS items have been translated from English into Norwegian, and back-translated into English by members of our research group (16).

#### Other baseline measures

Parents were asked to provide the date of birth and gender of the child, and whether she or he was born in Norway.

#### Measures of parents’ socio-demographics

Parents’ marital status was assessed with six response options: *single*, *married*, *cohabiting*, *separated*, *divorced* or *other*. The questionnaire asked about the highest completed education of both parents, with five response alternatives: *less than 9 or 10 years of primary school*, *primary school*, *secondary school or high school*, *university 4 years or less* or *university more than 4 years*. The work situation of the one parent who answered the questionnaire was assessed with the following response alternatives: *work full-time*, *work part-time*, *‘housewife’, sick leave*, *leave*, *disabled*, *occupational rehabilitation*, *student*, *unemployed* or *other work situation*. In addition, parents entered their gender and their own age in years. Non-Norwegian descent of both parents was approximated by the question of whether they were born in Norway. Parents reported their own weight in kilograms and height in centimetres.

#### Intervention compliance

Pedagogical leaders were asked to score the degree of compliance with the intervention elements (warm lunches [both intervention groups] and sensory education [only group 2 diet + Sapere]) from 1 (‘very small degree’) to 10 (‘very large degree’) or 0 (‘not completed’). The individual scores were added and divided by number of times assessed, leading to a mean score for each element. Mean score for the warm lunches was 9.1 (SD = 0.9), with a range from 6.8 to 10. Mean score for the sensory education was 8.8 (SD = 1.2), ranging from 6.3 to 10. No assessment was made to measure whether the children ate the food they were served.

No assessment was made for web use; however, all information was digital, meaning that kindergarten staff had to use the web-based information to conduct and record the intervention elements.

### Statistical analysis

Since the outcomes were collected from a self-reported questionnaire, there was some loss to follow-up, meaning that a full intention-to-treat (ITT) analysis could not be performed (58). However, the ITT principle was followed in spirit, and those with outcome data were analysed according to the group they were allocated to irrespective of adherence. All analyses were done on the complete cases since no new information can be gained from multiple imputation when only the outcome data are missing, and there are no available auxiliary variables related to the missingness (59). However, to address any imbalances that may have resulted from the cluster design and losses to follow-up, we also present a set of adjusted effect estimates, controlling for the baseline values of each outcome, and maternal and paternal education.

Baseline characteristics of the three groups (control, diet and diet + Sapere) were compared using descriptive statistics. To understand the potential for bias caused by losses to follow-up, these statistics were calculated in the entire sample, in those loss to follow-up and in the complete cases (analysis sample) (60). Descriptive data are presented as mean (SD) or median (IQR) as appropriate, depending on their distribution.

Negative binomial models were fitted to estimate the effect of the intervention on the count outcomes of total vegetable intake per day and intervention vegetable intake per week. The per week scale was chosen for intervention vegetables because the count was low.

For the binary total vegetable intake (≥17.5 portions per week) and total intervention vegetable intake (≥1 per week) outcomes, Poisson regression was performed. Poisson was preferred over logistic regression because the outcomes were relatively common, and hence, in this scenario, risk ratios are much easier to interpret than odds ratios.

Linear regression was used to estimate the intervention effects on the child food neophobia score (CFNS). For all inferential analyses, standard errors were corrected for the cluster design with a robust estimator. To check the robustness of the findings to the choice of Poisson model for the binary vegetable intake outcomes, the analyses were repeated using a logistic model. Findings were similar (results available on request). Since CFNS was highly skewed and a log transformation had little effect on its shape, to check the robustness of the CFNS results, we also fitted an ordinal logistic regression model as a sensitivity analysis. This was done to remove the influence of high observations, splitting the outcome into three CFNS groups (<10, 10–19 and 20+), where the middle group approximately captured the middle 50% of the sample at baseline.

Data analyses were performed using SPSS Statistics, versions 24.0 and 25.0, and Stata version 15.1.

## Results

### Study sample

**Fig. 1:**
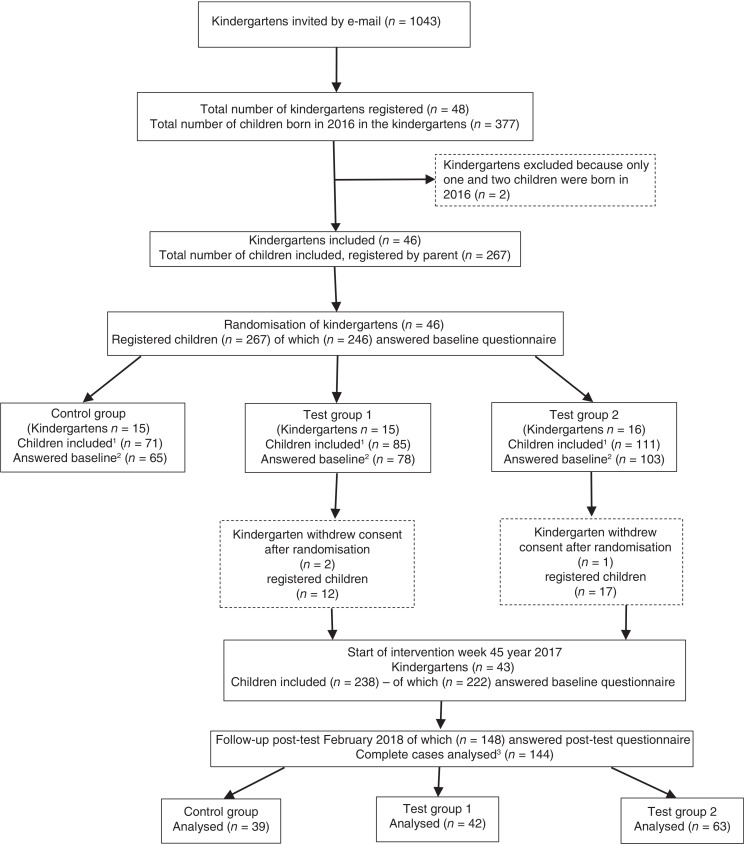
Flowchart of the trial. ^1^Children registered for the study by a parent. ^2^Answered baseline questionnaire. ^3^Children whose parent completed questionnaires at both baseline and post-intervention.

Out of the 48 kindergartens that registered for the study, two kindergartens were excluded before randomisation because they had fewer than three children born in 2016, leaving 46 kindergartens that were cluster randomised (Fig. 1). In total, 267 children were registered for the study. Twenty-one parents registered for the trial but did not complete the baseline questionnaire, leaving 246 children. Three of the kindergartens (*n* = 29 children) withdrew consent shortly after randomisation (two of them due to sick leaves and pregnancies amongst the staff and one kindergarten withdrew due to economic issues). Seventy-three parents (34%) did not complete the post-intervention questionnaire, leaving 144 children for the main analysis (total loss to follow-up: 102/246 = 41%) (Fig. 1).

Table 2 presents baseline characteristics of the total sample, and baseline comparisons of the randomised groups, those lost to follow-up and the complete cases to be analysed. Of the children with completed baseline data (*n* = 246), all were born in Norway, 47.6% were girls and the mean age was 16.7 months. Median intake of vegetables at baseline was 19.2 times per week, and median intake of the three intervention vegetables was 0.1 times per week. Mean score on the CFNS was 14.3 (SD = 7.1).

**Table 2:** Baseline characteristics of all participants with answered baseline questionnaire, those lost to follow-up and complete cases

Baseline variable	Total (all participants answered baseline)	All participants answered baseline (*N* = 246)			Lost to follow-up (*N* = 102) (did not complete post-intervention questionnaire)			Remaining participants/complete cases (*N* = 144)		
	*N* = 246	Control (*n* = 65)	Group 1(diet)(*n* = 78)	Group 2(diet + Sapere) (*n* = 103)	Control(*n* = 26)	Group 1(diet) (*n* = 36)	Group 2 (diet + Sapere) (*n* = 40)	Control (*n* = 39)	Group 1(diet) (*n* = 42)	Group 2 (diet + Sapere) (*n* = 63)
Number of kindergartens	48	15	16	17	12	14	12	14	14	15
Mean age (months) (SD)	16.7 (3.0)	17.1 (3.2)	16.5 (2.9)	16.5 (2.9)	17.7 (3.0)	16.9 (3.0)	16.6 (3.1)	16.8 (3.4)	16.3 (2.9)	16.4 (3.0)
Gender female, *N* (%)	117 (47.6)	33 (50.8)	39 (50.0)	45 (43.7)	14 (53.8)	12 (44.4)	13 (52.0)	19 (48.7)	24 (57.1)	28 (44.4)
Ethnicity child born in Norway (%)	100	100	100	100	100	100	100	100	100	100
Parent^a^										
Mean age (years) (SD)	30.9 (5.4)	30.9 (7.2)	30.6 (5.1)	31.1 (4.4)	30.4 (7.8)	31.6 (4.9)	32 (5.5)	31.1 (6.8)	30.2 (5.5)	31.4 (4.0)
Gender female, *N* (%)	219 (88.7)	60 (92.3)	65 (82.3)	94 (91.3)	24 (92.3)	21 (77.8)	22 (88.0)	36 (92.3)	36 (85.7)	57 (90.5)
Mean BMI (kg/m^2^)	25.4 (4.4)	26.0 (4.2)	25.0 (4.5)	25.4 (4.4)	23.3 (4.6)	25.0 (4.9)	24.9 (5.0)	26.3 (4.0)	25.3 (5.1)^1^	25.6 (4.4)
Parents living together (%)	94.3	93.8	92.4	96.1	25 (96.2)	23 (85.2)	23 (92.0)	36 (92.3)	40 (95.2)	61 (96.8)
Ethnicity mother born in Norway, *N* (%)	226 (91.5)	58 (89.2)	72 (91.1)	96 (93.2)	21 (80.0)	25 (92.6)	22 (88.0)	37 (94.9)	38 (90.5)	60 (95.2)
Ethnicity father born in Norway, *N* (%)	221 (89.5)	59 (90.8)	72 (91.1)	90 (87.4)	23 (88.5)	25 (92.6)	23 (92.0)	36 (92.3)	39 (92.9)	55 (87.3)
Mothers’ education high,^b^*N* (%)	158 (64.0)	37 (56.9)	53 (62.4)	68 (61.3)	9 (34.6)	13 (48.1)	14 (56.0)	28 (71.8)	30 (71.4)	46 (73.0)
Fathers’ education high,^b^*N* (%)	104 (42.1)	27 (41.5)	37 (46.8)	40 (38.8)	11 (42.3)	10 (37.0)	12 (48.0)	16 (41.0)	20 (47.6)	20 (31.7)
Child vegetable intake (total)										
Median times per week (IQR^2^)	19.2 (12.6–26.0)	18.7 (11.2–23.4)	17.6 (12.4–26.8)	20.5 (13.3–28.5)	18.1 (7.7–22.7)	18.3 (13.2–25.5)	21.1 (17.4–30.8)	19.6 (12.8–24.9)	17.1 (11.4–27.4)	18.1 (11.1–26.6)
≥2.5 times per day (≥17.5 per week) – *N* (%)	139 (56.5)	36 (55.4)	40 (51.3)	63 (61.2)	13 (50.0)	19 (52.8)	30 (75.0)	23 (59.0)	21 (50.0)	33 (52.4)
Child intake of intervention vegetables^c^										
Median times per week (IQR^2^)	0.1 (0.0–0.5)	0.0 (0.0–0.3)	0.1 (0.0–0.7)	0.1 (0.0–0.5)	0.0 (0.0–0.3)	0.1 (0.0–0.3)	0.1 (0.0–0.7)	0.1 (0.0–0.2)	0.3 (0.0–1.1)	0.1 (0.0–0.5)
≥1 weekly – *N* (%)	42 (17.0%)	7 (10.8%)	18 (23.1%)	17 (16.5%)	3 (11.5%)	6 (16.7%)	7 (17.5%)	4 (10.3%)	12 (28.6%)	10 (15.9%)
Child food neophobia score (range 6–42)										
Mean (SD)	14.3 (7.1)	14.9 (6.5)	13.7 (6.7)	14.3 (7.9)	16.5 (6.3)	14.3 (6.6)	15.7 (8.3)	13.9 (6.5)	13.1 (6.8)	13.5 (7.5)
Median (IQR^2^)	12 (8–18)	14 (10–19)	12 (8–17)	12 (8–18)	18 (12.5–20)	12 (10–17)	12 (9.25–23.5)	12 (9–18)	12 (7–17)	11 (7–18)

a The parent who filled out the baseline questionnaire, ^1^1 missing.

b Education high = university or college, ^2^IQR = interquartile range (25th to 75th percentile).

c Intake of spinach, fennel and celeriac in total.

The cluster randomisation produced groups that were broadly comparable at baseline. Mothers without a higher education were more likely to drop out of the study. Amongst the complete cases, there were slightly fewer fathers with a higher education in group 2 (diet + Sapere), and a slightly higher baseline intake of intervention vegetables amongst children in group 1 (diet). To understand whether these imbalances biased our effect estimates, the adjusted models control for these variables.

The main analyses that estimate the intervention effects are presented in Tables 3–5. Table 3 presents estimates for the effect of the intervention on the number of vegetables consumed per day and the number of intervention vegetables per week. The results from the unadjusted analysis suggest that those in group 1 (diet) consumed on average 24% (95% confidence interval [CI]: 0 to 52%) more portions of vegetables per day compared to the control group (*P* = 0.046). Adjusting for baseline vegetable intake and parental education only slightly attenuated this estimate (*P* = 0.068). The results also suggest that both group 1 (diet) and group 2 (diet + Sapere) consumed on average, three to four times more intervention vegetables per week compared to the control group. After adjustment for baseline intake of intervention vegetables and parental education, this effect was still evident for the diet + Sapere intervention (group 2, *P* = 0.02) but was attenuated and no longer statistically significant for the diet only intervention (group 1).

**Table 3:** Estimates for the effect of the intervention on the frequency of total vegetable intake per day and intervention vegetables per week expressed as incidence rate ratios (IRRs)

Outcome	Main (unadjusted analysis)		Secondary (adjusted analysis)*	
	IRR (95% CI)	*P*	IRR (95% CI)	*P*
**Total vegetable intake (per day)** Control Group 1 (diet) Group 2 (diet + Sapere)	Ref. 1.24 (1.00–1.52) 1.20 (0.92–1.57)	0.046 0.171	Ref. 1.20 (0.98–1.47) 1.14 (0.93–1.39)	0.068 0.217
**Intervention vegetable intake (per week)** Control Group 1 (diet) Group 2 (diet + Sapere)	Ref. 3.96 (1.62–9.72) 3.10 (1.22–7.84)	0.003 0.017	Ref. 1.80 (0.78–4.13) 2.63 (1.14–6.05)	0.166 0.020

* Adjusted for baseline intake of outcome, maternal and paternal education.

Table 4 presents the effect estimates for meeting thresholds of total vegetable intake in accordance with national recommendations and for a threshold of intervention vegetable intake. There was no evidence for an effect of the intervention on the likelihood of consuming ≥2.5 servings of vegetables per day (≥17.5 times/week). Adjusting for baseline vegetable intake also made little difference to this result. There was some evidence that the intake of the three intervention vegetables was higher in the intervention groups compared to the control group (Table 4). Children in group 1 (diet) were 4.6 times more likely to consume the intervention vegetables at least once a week compared to children in the control group (RR 4.64, 95% CI: 1.2 to 17.5, *P* = 0.02), and children in group 2 (diet + Sapere) were 3.3 times more likely to consume the intervention vegetables at least once a week compared to children in the control group (95% CI: 0.8 to 13.1, *P* = 0.09). After adjusting for the baseline differences in intervention vegetable intake and parental education, these effects were attenuated and the statistical evidence no longer there.

**Table 4:** Estimates for the effect of the interventions on the probability of having vegetable intake in accordance with national recommendations (all vegetables) and intervention vegetables at least once a week, expressed as relative risks (RRs)

Outcome	Main (unadjusted analysis)		Secondary (adjusted analysis)*	
	RR (95% CI)	*P*	RR (95% CI)	*P*
**Vegetable intake (≥17.5 per week)** Control** Group 1 (diet) Group 2 (diet and sensory)	Ref. 1.06 (0.76–1.46) 1.01 (0.66–1.56)	0.74 0.95	Ref. 1.11 (0.83–1.50) 1.03 (0.71–1.48)	0.46 0.88
**Intervention vegetables (≥1 per week)** Control** Group 1 (diet) Group 2 (diet and sensory)	Ref. 4.64 (1.23–17.5) 3.30 (0.83–13.1)	0.023 0.09	Ref. 3.08 (0.84–11.3) 2.85 (0.77–10.46)	0.091 0.12

* Adjusted for baseline value of outcome, maternal and paternal education.

** The control group is the reference group, RR = 1.0.

### Food neophobia

Table 5 presents estimates for the effect of the interventions on CFNS. There was a weak suggestion that children in group 1 (diet) had a lower CFNS compared to the control group after the intervention with the mean difference of 2.5-points (*P* = 0.055). However, there was no evidence after adjusting for baseline CFNS and parental education. There was also no evidence for an effect in group 2 (diet + Sapere) on the level of food neophobia. In the sensitivity analysis (Supplementary Table 1) using three categories of CFNS and ordinal logistic regression, there was also no evidence for an effect of either of the interventions on the level of food neophobia.

**Table 5:** Estimates for the effect* of the interventions on child food neophobia score (CFNS)

Outcome	Main (unadjusted analysis)		Secondary (adjusted analysis)^a^	
	Mean diff. vs control group (95% CI)	*P*	Mean diff. vs control group (95% CI)	*P*
**Control** Group 1 (diet) Group 2 (diet + Sapere)	Ref. −2.5 (−5.1 to 0.1) −0.7 (−4.4 to 2.9)	0.055 0.69	Ref. −2.0 (−4.5 to 0.6) −0.5 (−2.7 to 1.7)	0.12 0.67

* From a linear regression.

a Adjusted for baseline value of outcome, maternal and paternal education.

## Discussion

The results of this study suggested that children in both intervention groups had a higher intake of the intervention vegetables after the intervention, but with evidence only for group 2 (diet + Sapere) in the adjusted analysis. We also found a weak suggestion that the diet intervention may increase total vegetable intake, but the results were inconclusive. Our study was unable to detect any effect for either intervention group on the level of food neophobia.

A recent meta-analysis (18) revealed that interventions implementing repeated taste exposure of vegetables had better effects than those which did not. The authors of this meta-analysis concluded that 8–10 exposures should be recommended to achieve an increase in intake in children aged 2–5 years. However, several intervention studies have suggested that as little as three to five exposures to a novel vegetable increases intake of the target vegetable in young children, and that the youngest children require less exposure than the older children (19–21). In our trial, participants were offered at least six exposures of each of the intervention vegetables. There is some evidence that the effect of repeated exposure on acceptability is likely to generalise to other foods within the same food category (21, 61–63). We can only speculate whether an increased number of exposures of the intervention vegetables would have increased the total vegetable intake in the participating children.

A recent systematic review of methods for increasing vegetable consumption in early childhood suggests that repeated exposure is a highly effective method for increasing children’s vegetable consumption, which may benefit from being paired with modelling by peers of parents (64). In our trial, the repeated exposures were paired with social factors such as modelling by peers and kindergarten staff. In addition, intervention group 2 received sensory lessons, whilst their parents and kindergarten staff had access to information on relevant subjects such as food neophobia, repeated exposure and role modelling. Multicomponent interventions, like this trial, may have the potential of yielding positive results (65). The results suggested a higher intake of the intervention vegetables in group 2 (diet + Sapere); however, there were no indication that intervention group 2 had superior compliance with vegetable recommendations relative to the control group than intervention group 1 (diet). The results on total vegetable intake seemed to favour group 1 (diet), but the effect sizes and CIs for the two intervention groups were quite similar and made it difficult to conclude. This could be due to the lack of statistical power because of the large drop-out.

The Sapere method is used in both kindergartens and schools in some countries (27). During the last decade, some research has shown that allowing children to touch, smell and play with new food makes pre-schoolers more willing to try and taste them (26, 66, 67). The higher intake of the intervention vegetables in group 2 could be caused by the Sapere sensory lessons, which perhaps made the children more curious about different and novel vegetables. It is also possible that the parents in group 2 were more aware of the use of fennel, spinach and celeriac because of the focus on these vegetables during the intervention period. A change in behaviour as a response to observation and assessment is known as the Hawthorne effect and may have implications for the generalisability of results (68, 69).

In a recent review, the authors argue that sensory lessons do not appear to greatly affect food preferences, but some studies found a decrease in food neophobia, at least in the short term (70). However, the studies referenced in this review were all performed in school-aged children. To our knowledge, there are no other intervention studies on child food neophobia that have targeted children before the onset of food neophobia, normally around the age of 2 years. Helland et al. (16) found a mean score on the CFNS of 18.2 (SD = 9.3) amongst toddlers with a mean age of 28 months. In our sample of children with a mean age of nearly 17 months at baseline, the mean CFNS was 14.3 (SD = 7.1), which supports the perception that food neophobia increases during the period from 2 years and further (12, 71). We hypothesised that children in intervention group 2 (diet + Sapere) would have a lower increase in CFNS than group 1 (diet) due to the sensory education provided in group 2. However, we were not able to detect any difference from the control group in either intervention group. The relatively short intervention period of 3 months may have made it difficult to detect a difference in the development of food neophobia.

A strength of our study concerns the diversity of the recruited kindergartens. The 43 kindergartens that participated in the study were from four counties in different parts of Norway, both large and small. The sample included private and public kindergartens from both urban and rural areas, so it is probable that our sample is representative for kindergartens in Norway demographically. The fact that the parents were only asked to complete questionnaires whilst the kindergarten staff had to do the tasks necessary to implement the intervention may have reduced a potential selection bias attributable to participant burden. Second, the intervention was conducted in a natural setting, making it conceivable that the intervention can be implemented in kindergartens throughout the country with the internet-based administration approach. This factor makes it easy for kindergarten staff and parents to find and use the recipes and tools. Third, in planning this intervention, we focused on uncomplicated dishes, so that kindergarten staff with relatively low cooking skills could manage to carry out the intervention menus. Cooking activities, especially cooking novel food dishes, in kindergarten can be challenging, especially in kindergartens that do not have their own kitchen staff, a situation that is quite common in Norway (32). Fourth, Johannessen et al. found that kindergarten staff experienced the Sapere method successful as an educational tool amongst toddlers, but that three times a week was too often (32). In our study, the sensory lessons were conducted once a week, a frequency that may be more feasible to implement.

There are several limitations of our study that need consideration. First, recruitment of kindergartens and parents turned out to be quite difficult. According to our sample size calculation, which is somewhat limited as we had to estimate the ICC, we needed at least 58 participants in each group for the effect analysis. When the intervention in the kindergartens started, we had baseline data for 246 children, which exceeded the target of 210 children estimated by the original sample size calculation. However, the loss to follow-up was larger than expected because many parents did not complete the follow-up questionnaire. We only used email reminders (*n* = 2) as retention strategy. There might have been fewer lost to follow-up had we also included strategies such as monetary incentives (72). The relatively large loss to follow-up meant loss of statistical power, and our findings remain inconclusive for the total vegetable intake outcome. Larger studies are, thus, warranted to secure statistical power to detect true effects, and alternative methods should be considered to avoid large loss to follow-up. Second, we do not have information about why the non-participating kindergartens chose to refuse to participate in the study. The participating kindergartens may have been more interested in diet and health than those who refused to participate, causing a potential selection bias. Also, the sample of participating parents was relatively homogeneous – the majority were highly educated mothers of Norwegian ethnicity, which certainly limits generalisability to other Norwegian ethnic groups. Nonetheless, the education level in Norway is high, so the sample of mothers at baseline was quite representative for the general female population in Norway, with 63.9% of the mothers being highly educated (university or college), compared to 59.6% of women in the age between 30 and 34 years in the general population (73). Third, the findings of the study are based on parents’ self-report, which may have its weaknesses. Self-reported data entail a risk of social desirability bias, both in the form of over- and under-reporting. There are also limitations regarding the questionnaire used to assess food intake. The questionnaire does not measure absolute food intake, only frequency of intake. In our study, the frequency of vegetable intake at baseline was high (a median of almost three times per day). This can probably be a correct measure of frequency of vegetable intake during the day, but the amounts eaten of each vegetable most likely do not correspond to three full vegetable portions per day, which, in fact, is higher than the recommended intake of vegetables. It is possible that high-frequency users consume very small amounts each time, and the opposite, that low-frequency users consume larger amounts each time. Hence, we cannot exclude the possibility that the intervention resulted in higher total intake of vegetables through increased portion sizes without affecting the frequency of intake to the same degree. Additionally, it can be difficult for parents to report their child’s food intake since the child eats many of his/her meals in kindergarten. However, as most children eat food brought from home, and the fact that it is quite common for parents in Norwegian kindergartens to be informed about what the children eat during the day, we believe that parents have an overview of their child’s diet. This should not be any different for the control and intervention kindergarten parents and cannot explain the changes observed. Furthermore, the validation study of the original version of the FFQ for 2-years-olds indicated that even if the children are staying in kindergarten, the parents seem to be able to report the diet of their child (51). FFQs are frequently used because they are simple, quick and reliable tools compared with other more time-consuming dietary assessment methods (74). We considered the FFQ suitable for use in our study since we primarily wanted it to measure vegetable variety and certain types of vegetables eaten, including vegetables that are eaten seldom, rather than amount of food or calories in the children’s diet.

Kindergartens are potentially important settings for influencing children’s food choice and habit formation at an early age, and there has been a call for intervention studies in this field (75). Web-based study programmes, like the one developed for the present study, have the potential to be both appealing, cost-effective and capable of reaching large groups of children, parents and kindergarten staff. However, that relies on kindergartens being willing and able to implement such programmes in their daily routines. Hence, there is a need to find out what resources the kindergartens need to be able to carry out similar projects. The weak evidence regarding the effects of the trial may have been caused not only by the low number of complete cases but also by the limited duration of the trial. Three months may be too short for a period to achieve the magnitude of effect that we aimed for. We believe that similar trials of longer duration could prove to be effective in improving both vegetable intake and level of food neophobia in young children.

## Conclusion

Our study suggests that a digitally delivered, dietary and sensory intervention conducted in kindergartens can promote the intake of intervention-targeted vegetables. We also found a very weak suggestion that the diet intervention may increase total vegetable intake, although this requires more investigation. Our study was unable to detect any robust effects for either intervention group on the level of food neophobia. The results suggest that similar scalable web-based diet and food sensory interventions amongst 1-year-olds may have utility as a public health nutritional intervention, but future studies should implement procedures to mitigate losses to follow-up and may wish to consider a longer intervention period.

## Trial registration

ISRCTN98064772.
